# *Tribulus terrestris* Protects against Male Reproductive Damage Induced by Cyclophosphamide in Mice

**DOI:** 10.1155/2018/5758191

**Published:** 2018-08-28

**Authors:** Natasha Frasson Pavin, Aryele Pinto Izaguirry, Melina Bucco Soares, Cristiano Chiapinotto Spiazzi, Andreas Sebastian Loureiro Mendez, Fábio Gallas Leivas, Daniela dos Santos Brum, Francielli Weber Santos Cibin

**Affiliations:** ^1^Laboratório de Biotecnologia da Reprodução (Biotech), Campus Uruguaiana, Universidade Federal do Pampa, 97500-970 Uruguaiana, RS, Brazil; ^2^Faculdade de Farmácia, Universidade Federal do Rio Grande do Sul, Av. Ipiranga 2752, 90610-000 Porto Alegre, RS, Brazil

## Abstract

*Tribulus terrestris* (TT) has been considered as a potential stimulator of testosterone production, which has been related with steroidal saponins prevailing in this plant. Cyclophosphamide (CP) is the most commonly used anticancer and immunosuppressant drug, which causes several toxic effects, especially on the reproductive system. Patients who need to use CP therapy exhibit reduced fertility or infertility, which impacts both physically and emotionally on the decision to use this drug, especially among young men. We hypothesized that the treatment with TT dry extract would protect the male reproductive system against CP toxicity. Mice received dry extract of TT (11 mg/kg) or vehicle by gavage for 14 days. Saline or CP was injected intraperitoneally at a single dose (100 mg/kg) on the 14th day. Animals were euthanized 24 h after CP administration, and testes and epididymis were removed for biochemical and histopathological analysis and sperm evaluation. The dry extract of TT was evaluated by HPLC analysis and demonstrated the presence of protodioscin (1.48%, *w*/*w*). CP exposure increased lipid peroxidation, reactive species, and protein carbonylation and altered antioxidant enzymes (SOD, CAT, GPx, GST, and GR). Moreover, acute exposure to CP caused a reduction on 17 *β*-HSD activity, which may be related to the reduction in serum testosterone levels, histopathological changes observed in the testes, and the quality of the semen. The present study highlighted the role of TT dry extract to ameliorate the alterations induced by CP administration in mice testes, probably due to the presence of protodioscin.

## 1. Introduction

Cyclophosphamide (CP) is a cytotoxic bifunctional alkylating agent that belongs to the class of nitrogen mustards. It is used for the treatment of chronic and acute leukemia, multiple myeloma, lymphomas, and rheumatic arthritis and in the preparation for bone marrow transplantation [1, 2]. A single injection of a high dose of cyclophosphamide during the development of mouse spermatocytes resulted in heritable translocations [3], and the male cancer patients treated with cyclophosphamide exhibited an increased incidence of oligospermia and azoospermia [4].

It has been reported that acrolein, one of the metabolites of CP, is one of the main responsible for the increased production of reactive oxygen species (RS) and lipid peroxidation, which is one of the possible mechanisms responsible for its toxicity [5]. Oxidative stress is a key factor in the etiology of male infertility, since increased testicular oxidative stress can cause changes in the dynamics of testicular microvascular blood flow and endocrine signaling leading to an increase in germ cell apoptosis and subsequent hypospermatogenesis [6]. There are some papers regarding the benefits of antioxidants in protecting the male reproductive system from deleterious effects of reactive oxygen species caused during CP exposure [7, 8].

*Tribulus terrestris* L. (TT) is a member of the *Zygophyllaceae* family. It is widely distributed in Africa, western Asia, China, Japan, Korea, and Europe. This plant is known by several common names: puncture vine, caltrop, goat head, bull's head, ground burr nut, devil's thorn. *Tribulus terrestris* has been used in folk medicine throughout history to treat conditions such as impotence, rheumatism, edema, hypertension, and kidney stones [9, 10]. Although experimental and clinical studies partially confirm some effects of TT on libido and sperm production, there are still a lot of debates about the possible mechanisms of action, as well as its therapeutic application. Gauthaman et al. [11] showed that castrated rats treated with TT significantly increased the levels of the hormone testosterone in comparison to noncastrated rats. Thus, the authors suggest that this may also be reflected in humans during andropause, through the decline in the levels of sex hormones such as testosterone. It is believed that TT strongly affect the androgen metabolism, significantly increasing levels of testosterone or its precursors, and some studies indicate that this effect is due to the dominant component of the TT: protodioscin. Authors have demonstrated that for both castrated and noncastrated rats and for men with low testosterone level, there was an increase of this hormone through the use of TT or isolated protodioscin, thus demonstrating the potential of this plant as well as its dominant component in the improvement of male hormone levels [11–13].

In a previous study, TT exerted a protective effect against testicular damage induced by cadmium in rats, and this effect seems to be mediated by its antioxidant action and it also stimulates the production of testosterone from the Leydig cells [14]. Other studies show that TT presented an antioxidant and protective effect in rats with heart disease and diabetes [15, 16].

Considering (a) the damage on the male reproductive system caused by CP and the relationship with oxidative stress, (b) the antioxidant potential of TT, and (c) the potential effect of TT in stimulating testosterone production, an effect directly related to the presence of protodioscin, the present study is aimed at evaluating the protective potential of TT dry extract on the male reproductive system of mice treated with CP.

## 2. Materials and Methods

### 2.1. Drugs and Chemicals

*Tribulus terrestris* dry extract (origin China) was purchased from Xi'an Green Life Natural Products Co., Ltd. (China), in November 2013, batch number: 20121023, CAS number: 18642-44-9. Fruits were used to prepare the dry extract (alcohol/water extraction was used according to the manufacturer). The steroid saponin concentration has been determined to be a minimum of 40% of the dry matter. Cyclophosphamide, protodioscin, and all other chemicals and reagents were purchased from Sigma Chemical Co. (St. Louis, MO, USA). Acetonitrile was purchased from Tedia (Fairfield, OH, USA). Acetic acid was obtained from Synth (São Paulo, Brazil). Purified water used on HPLC (high-performance liquid chromatography) analysis was prepared using Milli-Q Plus® (Millipore, Bedford, USA).

### 2.2. Animals and Treatments

Adult male Swiss albino mice (30–35 g) were used for this experiment. The animals were kept in an appropriate animal cabinet with forced air ventilation, in a 12-hour light/dark cycle, at a controlled room temperature of 22°C, with food (Puro Trato, RS, Brazil) and water ad libitum. The animals were used according to the guidelines of the Committee on the Care and Use of Experimental Animal Resources (Federal University of Santa Maria, Santa Maria, Brazil), and all efforts were made to reduce the number of animals used and their suffering. This study was approved by the Ethics Committee on the Use of Animals of Federal University of Pampa (protocol number 027/2015).

The animals were separated in four groups: control, CP, *Tribulus terrestris*, and CP + *Tribulus terrestris*. The dry extract of *Tribulus terrestris*, diluted in distilled water, was given orally and daily for fourteen days at the dose of 11 mg/kg bw [17]; corresponding to approximately the usual dose indicated for humans (750 mg/day and assuming a body weight of 70 kg), saline or CP (100 mg/kg bw) was administrated intraperitoneally only once, 1 hour after the last administration of dry extract of *Tribulus terrestris* (14 days). The dose of CP was selected based on earlier reports [18–21]. Animals were euthanized with pentobarbital (100 mg/kg bw) 24 h after CP administration, and the left testes were removed and homogenized in 50 mM Tris-HCl, pH 7.4 (1/10, *w*/*v*) and centrifuged at 2400*g* for 15 min. The supernatant (S1) was used for biochemical assays, and the right epididymis was removed in order to perform the evaluation of semen. Blood was collected by cardiac puncture with anticoagulant. Plasma was separated for enzyme immunoassays of testosterone. The right testes were fixed in Bouin's fixative (0.2% picric acid/2% (*v*/*v*) formaldehyde in PBS) for histological evaluation.

### 2.3. HPLC Analysis of Protodioscin

The analyses of extracts and the reference standard were performed using an Agilent® liquid chromatograph (Santa Clara, CA, United States) equipped with a model Q 1311A quaternary pump, ALS-G1329A autosampler, TCC-G1316A column oven, and G1315B photodiode array detector. ChemStation system software was used to control the equipment and to calculate data and responses from the LC system.

A reversed-phase Thermo Scientific Hypersil ODS C18 column (250 × 4.6 mm id, 5 *μ*m particle size) (Bellefonte, United States) was used. The mobile phase consisted of 0.025% acetic acid in water (solvent A) and acetonitrile (solvent B) with a flow rate of 1.0 mL/min, DAD detection at 250 nm, injection volume of 20 *μ*L, and column temperature at 25°C. A gradient elution was performed from 10% B to 90% B in 40 min and from 90% B to 10% B for the next 5 min, then it was kept at 10% B for 5 min. A calibration curve, with concentrations ranging from 100 to 1000 *μ*g/mL, was constructed from a standard solution containing protodioscin. The presence of this compound on extracts was confirmed by comparing it to the authentic standards and evaluating the chromatographic profile and UV absorption. All measurements and analysis were carried out in triplicate [22].

### 2.4. Biochemical Assays in Testes

Lipid peroxidation was determined by the formation of the thiobarbituric acid reactive species (TBARS) as described by Ohkawa et al. [23], in which malondialdehyde (MDA), one of the end products of peroxidation of fatty acids, reacts with thiobarbituric acid (TBA) to form a colored complex. MDA values are determined with the absorbance coefficient of the MDA-TBA complex at 532 nm = 1.56 × 10^5^ cm/mmol.

The levels of the reactive species (RS) were determined by a spectrofluorimetric method, using 2′,7′-dichlorofluorescein diacetate (DCHF-DA) assay [24]. An aliquot of S1 was incubated with DCHF-DA (1 mM). The oxidation of DCHF-DA to fluorescent dichlorofluorescein was measured for the detection of intracellular RS. The DCF fluorescence intensity emission was recorded at 520 nm (with 480 nm excitation) 30 min after the addition of DCHF-DA to the medium.

The formation of carbonyl groups, a parameter of oxidative damage to proteins, was measured based on the reaction of these groups with dinitrophenylhydrazine (DNPH), as previously described by Levine et al. [25]. Samples were incubated at laboratory temperature in the dark for 30 min, stirring at 15 min intervals. After centrifugation, the samples were washed three times with 1 mL of ethanol-ethyl acetate (1:1; *v*/*v*) to remove the residual DNPH reagent. The final precipitates were dissolved in buffer SDS 2% and placed in a water bath at 37°C for 10 min. The absorption of the reaction product was measured in a spectrophotometer at 370 nm. Results were expressed as nmol carbonyl/mg protein.

The activity of superoxide dismutase (SOD) was determined as described by Misra and Fridovich [26]. This method is based on the ability of SOD to inhibit the autooxidation of adrenaline to adrenochrome. The color reaction is measured at 480 nm. One unit of enzyme (1 IU) is defined as the amount of enzyme required to inhibit the rate of autooxidation of adrenaline to 50% at 26 ° C.

The catalase (CAT) activity was determined spectrophotometrically according to the method of Aebi [27], which involves monitoring the consumption of H_2_O_2_ in the presence of the sample (S1) (20 *μ*L) at 240 nm. Enzyme activity is expressed in units (1 U decomposes 1 *μ*mol H_2_O_2_/min at pH 7 and 25°C).

Glutathione peroxidase (GPx) activity was analyzed spectrophotometrically by the method of Paglia and Valentine [28]. GPx analysis was made by adding GSH, GR, NADPH, and a peroxide to begin the reaction, monitored at 340 nm as NADPH is converted to NADP^+^.

Glutathione reductase (GR) activity was determined as described by Carlberg and Mannervik [29]. In this assay, GSSG is reduced by GR at the expense of NADPH consumption, which is followed at 340 nm. GR activity is proportional to NADPH decay. An aliquot of S1 was added in the system containing 0.15 M potassium phosphate buffer (pH 7.0), 1.5 mM EDTA, and 0.15 mM NADPH. After the basal reading, the substrate (GSSG, 20 mM) was added. The enzymatic activity was expressed as nmol NADPH mmol/min/mg protein.

Glutathione S-transferase (GST) activity was analyzed spectrophotometrically at 340 nm, as described by Habig et al. [30]. The reaction mixture contained an aliquot of the homogenized tissue (S1), buffer sodium phosphate 0.1 M pH 7, GSH (100 mM), and 1-chloro-2,4-dinitrobenzene (CDNB) (100 mM), which was used as a substrate. Enzyme activity is expressed as nmol of CDNB conjugated/min/mg protein.

Glutathione (GSH) levels were measured by spectrophotometry. Homogenate was precipitated with 10% trichloroacetic acid (TCA) at 1:1 (*v*/*v*) proportion. After the centrifugation at 3000 rpm for 10 min, 0.5 mL of supernatant was added to 2 mL of 0.3 M Na_2_HPO_4_·2H_2_O solution. A 0.2 mL solution of dithiobisnitrobenzoate (0.4 mg/mL 1% sodium citrate) was added, and the absorbance at 412 nm was measured immediately after mixing it. The GSH levels were calculated using an extinction coefficient of 13,600 mol^−1^·cm^−1^ [31].

All assessments were measured at 25°C using a microplate reader (Hidex, Plate Chameleon).

### 2.5. 17*β*-Hydroxysteroid Dehydrogenase (17*β*-HSD) Activity

17*β*-HSD activity was assayed according to Jarabak et al. [32]. The supernatant fluid (200 *μ*L) was mixed with 950 *μ*L of 440 *μ*M sodium pyrophosphate buffer (pH 8.9), 250 *μ*L of bovine serum albumin (25 mg crystalline BSA), and 20 *μ*L of 0.3 mM 17 *β*-estradiol. The enzymatic activity was expressed as nmol NADH/min/mg protein.

### 2.6. Hormone Assays

Plasma testosterone (T) concentrations were determined using electrochemiluminescence (I-100 Architect, Abbott). The sensitivity of this assay was 0.02 ng/mL for T.

### 2.7. Analyses of Semen

#### 2.7.1. Sperm Motility and Vigor

For the evaluation of motility and vigor, the semen was diluted in 50 *μ*L of DMPBS (Nutricell) and analyzed subjectively, using a phase-contrast microscope at 100x magnification and objectively by semicomputerized system Sperm Class Analyzer (SCA), obtaining the value of the curvilinear velocity (VCL—*μ*m/s). These evaluations were performed by the same observer.

#### 2.7.2. Membrane Integrity

The membrane integrity was evaluated by the technique described by Harrison and Vickers [33] using a mixture of two probes propidium iodide (PI) and carboxyfluorescein diacetate [34]. After the incubation, 200 spermatozoa were placed under 400x magnification with epifluorescence illumination. Spermatozoa that fluoresced green throughout their length after staining with carboxyfluorescein diacetate were classified as being intact while all others were classified as damaged.

### 2.8. Histology

For histological studies, the right testis was fixed overnight in Bouin's fluid, dehydrated in ethanol, and embedded in paraffin. Tissue sections (5 *μ*m) were mounted on a glass slide and dried at 42°C for 24 h. The sections were then deparaffinized with xylene and rehydrated with alcohol and water. The rehydrated sections were stained with haematoxylin and eosin, mounted with 50% glycerol in PBS, and examined under a light microscope (400 and 1000x; Binocular, Olympus CX31, Tokyo, Japan) by a single, experienced examiner.

### 2.9. Protein Determination

Protein concentration was measured by the method of Bradford [35] using bovine serum albumin as the standard.

### 2.10. Statistical Analysis

The data were expressed as mean ± SD (*n* = 6). Statistical analysis was performed using one-way analysis of variance (ANOVA), and the differences between the means of experimental and control groups were analyzed statistically by Duncan's test (Statistical Software, 1999). A difference was considered significant at *P* < 0.05.

## 3. Results

### 3.1. HPLC Analysis of Protodioscin

**Figure 1:**
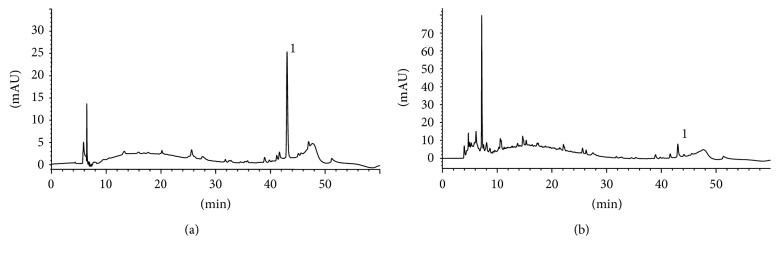
(a) Chromatogram of protodioscin reference standard at 500 *μ*g/mL in 70% hydroethanolic solution. (b) Chromatogram of aqueous extract (infusion) from TT, prepared on concentration of 0.056 g/5 mL. 1—Protodioscin retained at 43.03 and 42.99 minutes for reference standard solution and aqueous extract, respectively; detection at 250 nm.

Through HPLC analysis, quantifying the compound protodioscin in TT extract at a concentration of 166.24 *μ*g/mL was possible (Figure 1). This component was detected at retention time of 42.99 minutes.

### 3.2. Biochemical Assay

The group treated with CP showed a significant increase in the RS levels (28.86%), TBARS levels (55.83%), carbonyl levels (23.95%), SOD activity (16.03%), CAT activity (17.67%), GR activity (25.90%), and GST activity (62.20%) when compared to the control group. The TT dry extract treatment was effective in preventing these altered parameters. The group treated with CP + TT showed a significant increase in the GST activity (30.27%) when compared to the control group. GPx activity in group CP was decreased by 12.46% when compared to the control group, and the TT dry extract treatment had an increase of 19.96%. No alteration was observed on GSH levels in testes (Table 1).

### 3.3. 17*β*-Hydroxysteroid Dehydrogenase (17*β*-HSD) Activity

**Figure 2:**
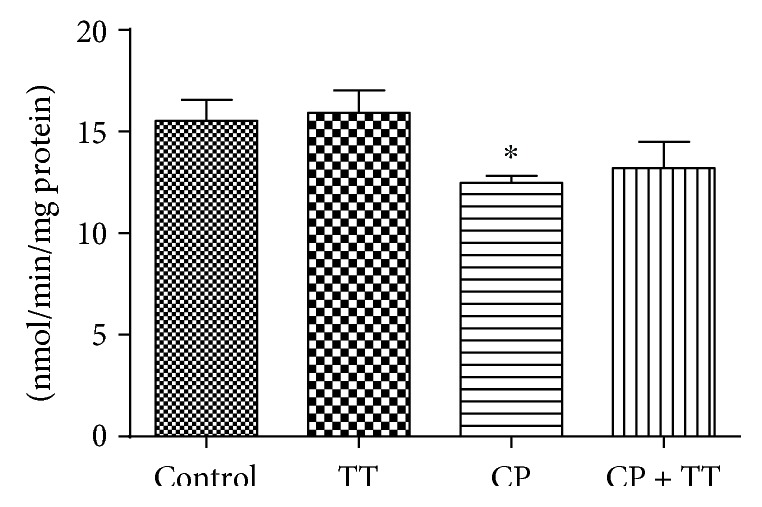
Effect of CP (100 mg/kg) and the dry extract of TT pretreatment (11 mg/kg) on 17*β*-HSD activity in testes. All the values are expressed as mean ± SD (*n* = 6). ^∗^*P* < 0.01, compared to the control group.

CP reduced the 17*β*-HSD activity in testes (19.70%). Animals that received concomitantly the dry extract of TT plus CP presented 17*β*-HSD activity statistically equal to control and CP groups, demonstrating a partial recovery from enzyme activity (Figure 2).

### 3.4. Testosterone Level

**Figure 3:**
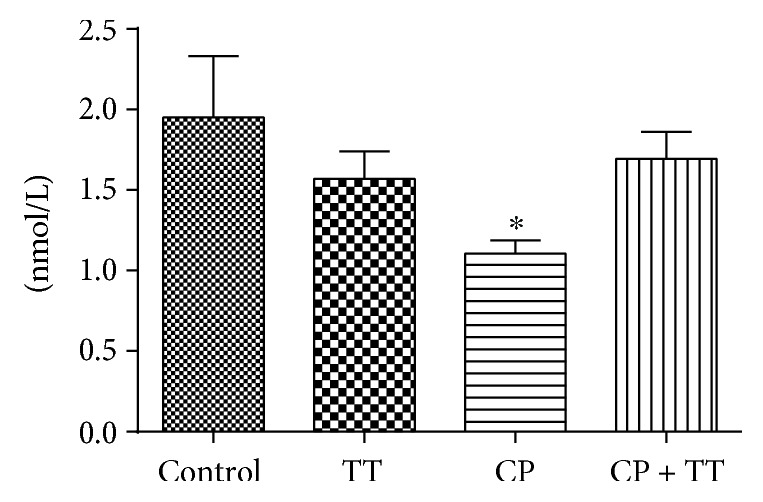
Effect of CP (100 mg/kg) and the dry extract of TT pretreatment (11 mg/kg) on serum testosterone (T) concentrations. All the values are expressed as mean ± SD (*n* = 6). ^∗^*P* < 0.01, compared with the control group.

The serum testosterone level in the group treated with CP showed a significant reduction (43.58%) when compared to the control group. TT therapy was effective in restoring this parameter (Figure 3).

### 3.5. Semen Analysis

The group treated with CP showed a significant decrease in sperm motility (41.07%), vigor (23.52%), integrity (43.44%), and VCL (25.43%), when compared to the control group. The dry extract of *Tribulus terrestris* treatment was effective in preventing this damage in sperm. The group treated with CP + TT showed a significant increase in motility by 53.57% when compared to the control group (Table 2).

### 3.6. Histology

**Figure 4:**
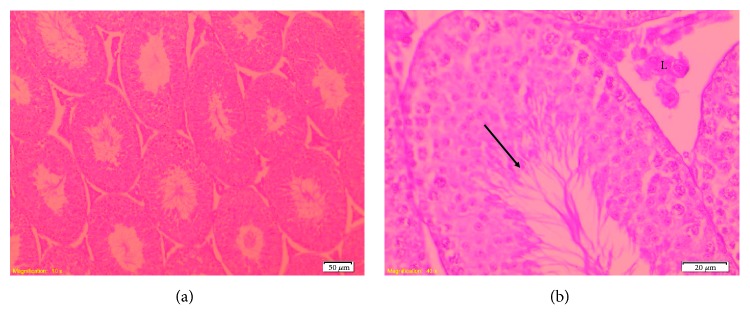
Photomicrograph of seminiferous tubules from mice in the control group. (a) Normal testicular architecture (100x). (b) Note the normal spermatogenic epithelium composed by different spermatogenic cells (arrow) and Leydig cells (L) (400x). HE staining.

Optical microscopic examination showed that in the control, TT, and TT + CP groups, testes had a normal testicular architecture with an orderly arranged spermatogenic and Sertoli cells. The spermatogonia and Sertoli cells were rested on the basement membrane of the seminiferous tubules. The Leydig cells with large and acidophilic cytoplasm were located in the interstitial tissue among seminiferous tubules (Figure 4).

**Figure 5:**
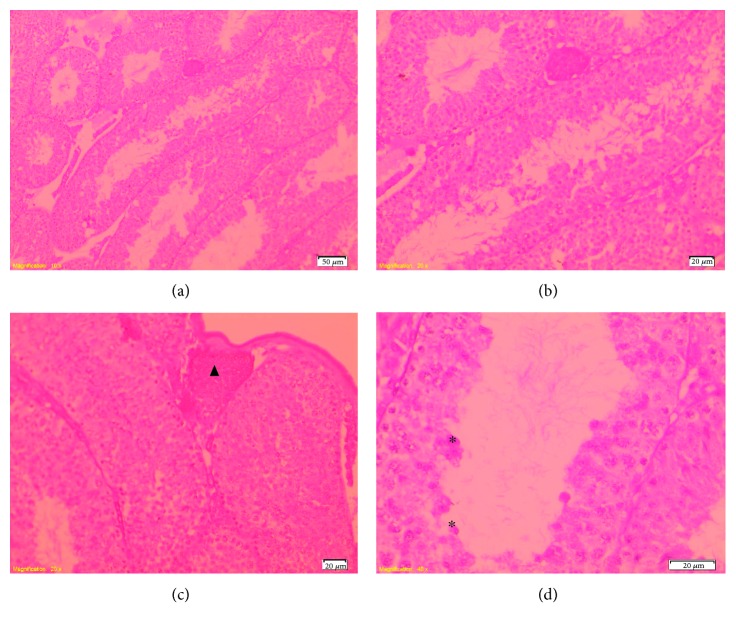
Photomicrograph of seminiferous tubules from mice treated with CP. (a)–(b) Disorganized seminiferous tubules (100–200x). (c) Note the congestion of the blood vessels (▲) (200x) and (d) exfoliated germ cells (^∗^) (400x). HE staining.

Mice treated with CP showed moderate disorganization of spermatogenic epithelium with germ cells present in the lumen of the seminiferous tubules. The Leydig cells in the interstitial spaces had a normal morphology. Regarding the interstitium, there was congestion of interstitial blood vessels (Figure 5).

## 4. Discussion

The present study demonstrated for the first time that the TT dry extract protects the male reproductive system of mice against damage induced by CP. CP is widely used in traditional medicine against various diseases, including cancer [36]; however, the adverse effects of this drug include reproductive toxicity and despite its great importance in therapy, the use of CP still raises many questions because infertility plays a critical role and impacts, both physically and emotionally, on the decision to use this medicine, especially among young people [4, 20].

Our data suggest that the exposure to a single dose of CP causes toxic effects on the male reproductive system, which are related to oxidative stress, by increasing lipid peroxidation, reactive species, and protein carbonylation as well as by altering antioxidant enzymes (SOD, CAT, GPx, GST, and GR), and the pretreatment with TT was effective in protecting against this assessed damage. It is known that the male reproductive system is a prominent target of free radicals, since the membrane of sperm is rich in polyunsaturated fatty acids and contains a low concentration of antioxidant enzymes. In this way, an imbalance in the production of ROS and antioxidant defenses during the process of cell division could reduce the production and quality of sperm, resulting in infertility [37, 38]. Currently, the use of natural antioxidants has been widely discussed in relation to its role in preventing damages to the reproductive system. Thus, Zanchi et al. [21] demonstrated that pretreatment for 14 days with green tea infusion (250 mg/kg) was partially effective in preventing the damage induced by CP exposure (100 mg/kg) on the reproductive system of male mice. In addition, this effect is possibly due to high concentrations of catechins and also due to the antioxidant activity of green tea infusion. In another study, Abd El Tawab et al. [39] showed that the *Satureja montana* extract (50 mg/kg/day) protected against testicular damage induced by CP (200 mg/kg) via antioxidative and antiapoptotic mechanisms.

17*β*-HSD is an enzyme that catalyzes the interconversion of androstenedione to testosterone. This enzyme plays an important role in the biological activity of steroid hormones such as estrogens and androgens. A reduction in its activity can decrease the serum testosterone level, spermatic count per testis, sperm count per epididymis, daily sperm production/gram testis, and sperm motility and a significant increase in abnormal sperm rates [32]. Evaluating our results, we found that acute exposure to CP caused a reduction in 17*β*-HSD activity, which may be related to the reduction in serum testosterone levels. In addition, these changes could be involved with the histopathological changes observed in the testis and the quality of the semen of these animals, since it is known that histopathological changes observed in this study can affect the maturation and sperm quality. These findings are in agreement with the literature, which demonstrate that cytotoxicity mediated by the use of CP in various doses tested and even in low doses has similar reproductive toxicity [20].

Many different compounds with a variety of biological properties and chemical structures have been identified from TT including steroidal saponins, phytosterols, phenolic compounds, tannins, terpenoids, amide derivatives, amino acids, and proteins [10]. However, literature data shows that steroidal saponins are responsible by major biological activities related to TT use. In fact, the steroidal molecular structure of the steroidal saponins such as protodioscin is thought to confer TT unique biological activities [11–13, 40].

Searching for an action mechanism, considering the beneficial actions of TT and according to studies related to the use of this plant both in animals and in humans [11, 13, 40], authors reported that protodioscin, the major substance of this extract, is likely to be responsible for these actions, since an improvement in sexual performance, muscle tonus, hormone levels and other benefits have been described in several studies. It has been demonstrated that TT containing protodioscin increases DHEA (dehydroepiandrosterone) levels in men. DHEA is an important steroid circulating in human plasma mainly synthesized by the adrenal glands and, to a lesser extent, by the gonads; it is a common precursor to both androgens and estrogens [7, 8].

**Figure 6:**
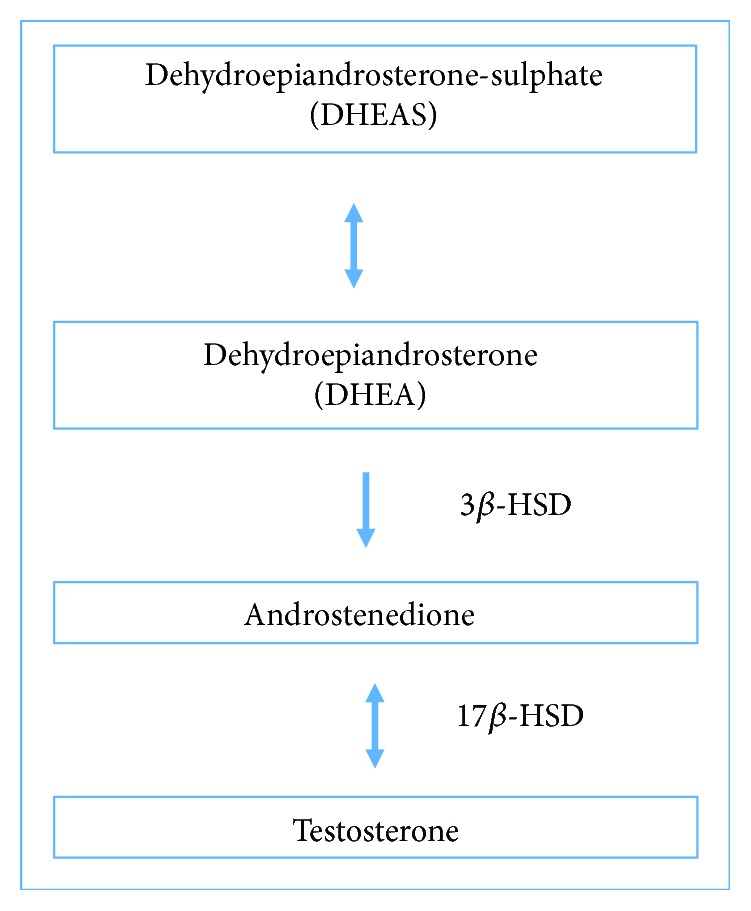
Correlation of hormone dehydroepiandrosterone sulphate (DHEA-S) with the levels of testosterone.

Adimoelja and Adaikan [12] showed that protodioscin TT improves male sexual function in diabetic and nondiabetic men treated with TT dry extract 750 mg/day for 3 weeks. The authors report that infertility may be related to low levels of DHEA in the blood values. We believe that the decrease in DHEA may also be associated with decreased levels of testosterone in serum and 17*β*-HSD activity. Thus, we believe that the protodioscin present in this dry extract of TT could possess the ability to increase serum testosterone levels and, although partially, 17*β*-HSD activity, which could improve sperm production (Figure 6).

In the present study, we verified that the dry extract used is rich in protodioscin (1.48%), similar to the values found by Dinchev et al. [22]. We cannot rule out the fact that other phytochemicals presented in TT dry extract could contribute to the observed effects. In fact, taking into account that TT presents many different compounds, it is possible that all of these substances are related to the useful effect observed in this study. But, considering the set of results, we believe that protodioscin is the main responsible for the beneficial effects found in this study, corroborating other findings in the literature [22, 40, 41].

## 5. Conclusion

The present study is the first to investigate the efficacy of dry extract of *Tribulus terrestris* to protect against testicular damage induced by cyclophosphamide. CP is the most commonly used anticancer and immunosuppressant drug, and patients who need to use CP therapy exhibit reduced fertility or infertility, which impacts both physically and emotionally on the decision to use this drug. The present study highlights the role of TT dry extract in ameliorating the biochemical parameters, analyses of semen, testosterone level, and histopathology alterations induced in mouse testes by CP administration. In fact, the protective role of TT in testicular CP-induced toxicity is evident, demonstrating to be a promising alternative, particularly in relation to its use in a patient requiring such therapy using cyclophosphamide. Although we cannot rule out the fact that other phytochemicals presented in TT dry extract could contribute to the observed effects, the results obtained in this study suggest that protodioscin present in this extract could be the main responsible for the beneficial effects visualized in our experiment. However, more studies are needed in order to understand the mechanism of TT dry extract in relation to its beneficial effects and possible interaction with anticancer drugs.

## Figures and Tables

**Table 1:** The effect of CP (100 mg/kg) and dry extract of TT (11 mg/kg) on biochemical assays in testes of mice.

	Control	TT	CP	CP + TT
RS (FU)	128.20 ± 17.06	142.20 ± 16.14^#^	165.05 ± 8.92^∗^	141.16 ± 9.04^#^
TBARS (nmol MDA/mg protein)	90.61 ± 6.62	88.32 ± 8.31^#^	141.20 ± 9.18 ^∗^	75.69 ± 0.68^#^
Carbonyl (nmol carbonyl/mg protein)	2.63 ± 0.37	2.78 ± 0.37	3.26 ± 0.69^∗^	2.96 ± 0.67
SOD (IU)	52.83 ± 3.52	51.86 ± 3.07^#^	61.30 ± 4.34^∗^	54.67 ± 3.75^#^
CAT (U Cat/mg protein)	0.95 ± 0.08	1.03 ± 0.074	1.12 ± 0.10^∗^	0.86 ± 0.05^#^
GPx (mmol/min/mg protein)	5.86 ± 0.46	5.79 ± 0.20^#^	5.13 ± 0.31^∗^	7.03 ± 0.19^∗^^#^
GR (nmol/min/mg protein)	25.05 ± 3.92	25.96 ± 2.15^#^	31.54 ± 2.44^∗^	29.10 ± 2.55
GST (nmol CDNB/mg protein)	814.80 ± 83.16	1137.57 ± 86.70^∗^^#^	1321.62 ± 88.65^∗^	1061.55 ± 115.02^∗^^#^
GSH (nmol GSH/g tissue)	269.89 ± 6.22	264.88 ± 9.39	270.35 ± 6.28	272.44 ± 8.29

All the values are expressed as mean ± SD. ^∗^*P* < 0.05 compared with group control. ^#^*P* < 0.05 compared with group CP.

**Table 2:** The effect of CP (100 mg/kg) and dry extract of TT (11 mg/kg) on epididymal sperm characteristics.

	Control	TT	CP	CP + TT
VCL (*μ*m/s)	81.70 ± 10.31	93.70 ± 17.77	60.92 ± 6.55^∗^	88.57 ± 3.52
Motility (%)	34.57 ± 6.42	43.40 ± 10.37	20.37 ± 5.35^∗^	53.23 ± 5.85^∗^^#^
Vigor (1–5)	4.25 ± 0.50	4.25 ± 0.50	3.25 ± 0.50^∗^	4.0 ± 0.01
Integrity (%)	36.83 ± 8.03	36.75 ± 10.25	20.83 ± 6.60^∗^	36.16 ± 5.96

All the values are expressed as mean ± SD. ^∗^*P* < 0.05 compared with group control. ^#^*P* < 0.05 compared with group CP.

## Data Availability

The data used to support the findings of this study are available from the corresponding author upon request.

## Abbreviations

CAT: Catalase
CDNB: 1-Chloro-2,4-dinitrobenzene
CP: Cyclophosphamide
DCHF-DA: 2′,7′-dichlorofluorescein diacetate
DNPH: Dinitrophenylhydrazine
EDTA: Ethylenediaminetetraacetic acid
GPx: Glutathione peroxidase
GR: Glutathione reductase
GSH: Glutathione
GST: Glutathione S-transferase
HPLC: High-performance liquid chromatography
MDA: Malondialdehyde
NADH: Nicotinamide adenine reduced
NADPH: Nicotinamide adenine dinucleotide phosphate reduced
17*β*-HSD: 17*β*-Hydroxysteroid dehydrogenase
RS: Reactive species
SCA: Sperm class analyzer
SOD: Superoxide dismutase
TBA: Thiobarbituric acid
TBARS: Thiobarbituric acid reactive species
TT: *Tribulus terrestris*
VCL: Curvilinear velocity.
